# Delay-Induced Transient Increase and Heterogeneity in Gene Expression in Negatively Auto-Regulated Gene Circuits

**DOI:** 10.1371/journal.pone.0002972

**Published:** 2008-08-13

**Authors:** R. Maithreye, Ram Rup Sarkar, Veena K. Parnaik, Somdatta Sinha

**Affiliations:** Centre for Cellular & Molecular Biology (CSIR), Hyderabad, India; Center for Genomic Regulation, Spain

## Abstract

A generic feature in all intracellular biochemical processes is the time required to complete the whole sequence of reactions to yield any observable quantity-from gene expression to circadian rhythms. This widespread phenomenon points towards the importance of time delay in biological functions. Theoretically time delay is known to be the source of instability, and has been attributed to lead to oscillations or transient dynamics in several biological functions. Negative feedback loops, common in biochemical pathways, have been shown to provide stability and withstand considerable variations and random perturbations of biochemical parameters. The interaction of these two opposing factors-of instability and homeostasis-are features that are widespread in intracellular processes. To test the effect of these divergent forces in the dynamics of gene expression, we have designed and constructed simple negatively auto-regulated gene circuits consisting of a basic regulator and transcriptional repressor module, and compared it with one, which has delayed repression. We show, both theoretically and experimentally, that delayed repression induces transient increase and heterogeneity in gene expression before the gain of stability effected by the negative feedback. This design, therefore, seems to be suitable for conferring both stability and variability in cells required for adaptive response to a noisy environment.

## Introduction

Networks of genetic and metabolic reactions, underlying intra-cellular processes, are interconnected multi-step chemical reactions having widely different time scales. The complex regulation of these metabolic and transcriptional networks is brought about by the interaction of simpler regulatory structures [1]–[4]. The two most important features that have engaged the attention of theoreticians and experimentalists in this area are-a) the role of stochasticity in regulating the precision in the output of such pathways, and b) the effect of regulatory designs in conferring different dynamical behaviour of the pathway constituents.

Much excitement has been generated in recent years to study how stochasticity/perturbation in the different reaction steps affects gene expression kinetics using both theory and experimental ‘forward engineering’ approach, based on the construction and analysis of artificial “gene circuits” for single genes, gene networks, and multi-step regulated pathways [see 5], [ for an excellent review and references; 6]–[13]. A similar approach has also been used to study and analyse simpler regulatory structures, which may provide some clues regarding the function of larger, more complex networks. The emphasis has been on finding the core regulatory designs that give rise to different dynamics (e.g., bistability, oscillations, etc) that are observed in intra-cellular processes such as the bistable genetic switch in λ phage [14], [15], ultradian and circadian oscillations in eukaryotic and prokaryotic organisms [16]–[17]. Though the positive feedback motif has been generally assigned for bistability [19]–[24], a variety of negative feedback processes have been proposed to underlie homeostatic and oscillatory dynamics [11], [21], [25]–[33].

Among the basic regulatory designs, feedback inhibition of gene expression is the most common motif in gene regulation, where the expression of a gene is down-regulated by either its protein product (*auto-regulation*), or by any other factor (*classical regulation*) [2], [34]. It is commonly understood, theoretically analysed, and experimentally demonstrated in the context of gene regulation, that a) negative auto-regulatory feedback provides a noise-reduction mechanism [5], [27], [35] leading to reduced heterogeneity in protein products in cell populations (but see 11 for the role of repression strength in noise reduction); and, b) negative auto-regulation is theoretically suggested to decrease the rise-time of gene expression [34], [36], which was experimentally demonstrated by the speeding up of transcription response in a simple negatively auto-regulated gene circuit [29]. This was also attributed as a reason for the predominance of transcription factors in *E. coli* being of the negatively auto-regulated type. From previous theoretical studies, negative auto-regulation has also been predicted to shift noise to higher frequencies, which is more easily filtered out by gene networks-a property conjectured to contribute to the prevalence of such auto-regulatory motifs in the regulation of 40% of *E. coli* genes [1], [37], [38].

One feature that is intrinsic to any intra-cellular biochemical process, such as transcription, translation, up and down stream regulation by gene products or metabolic pathway products, is the time required to complete the whole sequence of events. This points towards time-delay to be a ubiquitous factor in any intra-cellular process. Compared to the prokaryotes, the complexity of multi-stage regulation in eukaryotes, involving fast reactions (dimerization, protein–DNA binding/unbinding) and slow reactions (transcription, translation, degradation, multiple transcription factor cascades), is higher, and hence have been postulated to be a significant source of delay in the subsequent steps of the reaction [31], [39], [40]. These have been considered to be the underlying reason for oscillations in several periodic processes in eukaryotic organisms [5], [6], [31], [32]. Traditionally, along with nonlinearity, presence of additional steps in an auto-regulated pathway has been theoretically shown to induce oscillations [25], [26], [41], [42]. A gene circuit realisation of this was also shown to produce oscillations in an artificial three-regulator system [28].

Even though delay-induced instability is widely discussed in theoretical literature for negative feedback systems [41], [43], [44], a direct demonstration of the interaction of delay and negative auto-regulation using gene circuits has not been shown both experimentally and theoretically. What has been shown clearly is the role of noise and its consequent population heterogeneity in negatively auto-regulated circuits [27], [37], [45]–[51; see results reviewed in 5,11], faster response time of such circuits [29], presence of bimodality in cell populations [11], [52 even though both these involve classical negative regulatory circuits], and many examples of complex circuitry involving multiple regulatory steps giving rise to oscillations [31], [53]. Given the fact that time delay is known to induce instability, whereas, negative auto-regulation promotes stability and noise reduction, we put forward a very simple idea to compare two topologies of regulation–one, where the feedback by a repressor to its own production is immediate (Basic circuit), and the other, where the repressor is preceded by some other DNA sequence in the same transcriptional unit (Delay circuit). The hypothesis is that the repressor will take relatively longer time to be synthesized in the latter, and hence, the feedback process will be delayed when compared to the former. We propose that the comparison of the kinetics of gene expressions in these two model topologies would be indicative of the interaction of the two opposing factors-of instability and homeostasis–that are common features in intracellular processes. To address this issue, we constructed mathematical models, and equivalent negative auto-regulated gene circuits, to compare the effect of such topologies of regulation on the kinetics and the population heterogeneity of gene expression. Our results, from both deterministic and stochastic model simulations of the networks, indicate that delayed negative feedback can lead to an overshoot or pulse of gene expression, and a transient increase in the heterogeneity in concentrations of gene product among cells in a population, before the gain of stability is effected by the negative feedback. The experiments with simple gene circuits also show the overshoot and increase in population heterogeneity. Given the ubiquitous presence of delay in biochemical reaction pathways, along with predominance of negative auto-regulatory motif, this design, therefore, confers the potentially fitness-enhancing property of both stability and variability required for adaptive response.

## Results

**Figure 1:**
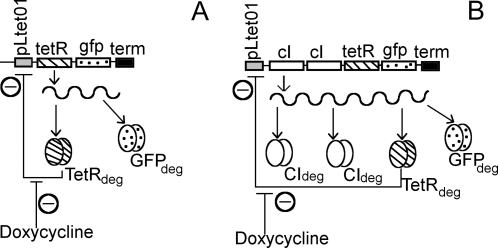
Design of negatively auto-regulated gene circuits: (A) Basic (TG), and (B) Delay (C2TG). See text for details.

In the following sections we discuss the theoretical and experimental results and compare them for the non-delayed (Basic) and the delayed negative feedback (Delay) circuits (described in Fig. 1).

### 1. Temporal dynamics of gene expression in the Basic and Delay circuits

#### a) Dynamics of the model gene circuits

**Figure 2:**
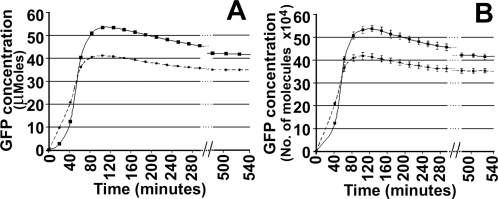
Theoretical kinetics of GFP: In Basic (*solid circles with dashed lines*) and Delay (*squares with solid lines*) circuits for (A) Deterministic model, and (B) Stochastic model (mean and standard deviation of 100 simulations).

The time course of Green Fluorescent Protein (GFP) in the Basic and Delay circuits for the four variable deterministic model and stochastic model (average of 100 simulations) are shown in Fig. 2 (A) and (B), respectively. The GFP concentrations in the Delay circuit in both models show a sharp increase (“overshoot”) followed by a gradual decline to the same steady state value (takes longer time than TetR due to slower degradation rate, as shown in Supporting Information S2, Fig. S9). The length of delay used here is one minute in addition to the basal value (Supporting Information S2). On increasing the length of delay, the extent of overshoot increases, but the steady state remains constant (not shown here). Thus, the introduction of delay that defers repression in the models circuits causes a transient pulse of gene expression. Overshoot in gene expression in the model Basic circuit can also occur due to increase in transcription-translation rates and plasmid copy number. For example, a ten-fold increase in translational efficiency and plasmid copy number over the basal values (β and g_t_ in Supporting Information S2), increases the overshoot by 57% and 10% respectively.

**Figure 3:**
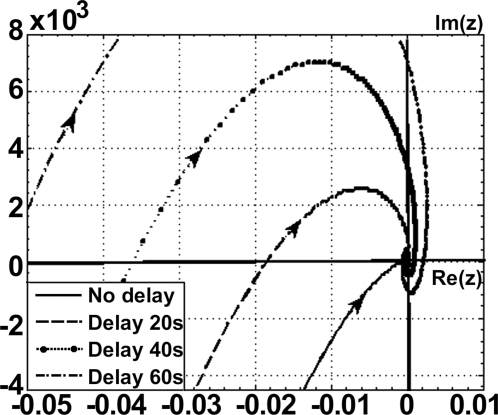
Nyquist loci for the deterministic model: Nyquist loci, G(iw), for the transfer function of the deterministic model for increasing delay. Arrows indicate the direction of increasing w.

Delays can make an otherwise-stable system less stable, i.e., the rate of decay of perturbations can decrease with an increase in the values of delay parameters, and a system, which has been otherwise non-oscillatory, can show oscillatory tendencies before converging to the steady state [43]. Such behaviour, especially when connected with global stability for all delays, is rare in literature. They imply that, even though periodic solutions cannot arise in such models due to delay, but the rate at which the solutions converge to the equilibrium state will depend on the size of the delay [54], [55]. Stability analysis of the deterministic model of both Basic and Delay circuits indicated that the system is globally stable. The Nyquist loci, G(iw), for the transfer function of the model is shown in Fig. 3 for a range of delay time-from 0 to 60 sec (Supporting Information S2, Table S1, for larger delay times). The analysis clearly shows that this model cannot show stable oscillatory behaviour under all delays considered, as there exists no closed curve around the origin. For all delay lengths the points on the negative real axis are encircled by the Nyquist loci, but the feedback gain, k (which is the negative of the inverse of the distance between the origin and the intersection of the curves at negative real axis) in all the cases are less than the critical value, k_c_ (k value calculated for zero-delay system). Thus, with increasing delay time, the system shows damped oscillations, and the steady state is reached with progressively larger excursion in the phase plane. Our theoretical analysis, therefore, predicts that our model gene circuits have the intrinsic property of transient “overshoot” dynamics with increasing delay.

The fact that both the deterministic and stochastic versions of the models show overshoot for the Delay circuit indicates that this feature is due to delay in repression and not solely due to intrinsic or extrinsic noise in the cell population. Hence from now onwards, we show results only from the stochastic model of the gene circuits.

#### b) Experimental studies

**Figure 4:**
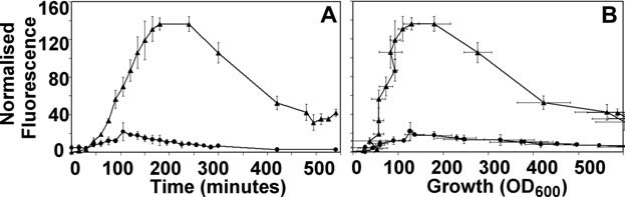
Experimental kinetics of GFP: In the Basic (*circles*) and the Delay (*triangles*) circuits upon induction in four independent experiments. (A) Normalised fluorescence versus time (minute); (B) Normalised fluorescence versus growth (OD_600_). Error bars (one standard deviation) are for both fluorescence and growth.

Both the Basic (TG) and the Delay (C2TG) circuits (Fig. 1) were induced with 25 ng/ml of Doxycycline, and Fig. 4 (A) and (B) show the normalised GFP fluorescence in the Basic (circles) and the Delay (triangles) circuits with time and with growth (OD_600_), respectively. The Basic circuit shows an initial increase in GFP expression, which decays towards a steady level over 9 hours. The Delay circuit, on the other hand, shows a large increase (overshoot) in fluorescence, which also decays towards a steady level, although this level is not the same as that reached by the basic circuit. We have done flow cytometric analysis to show that this is due to increased size of cells in the Delay circuit during growth at later time points, which show high fluorescence. A size correction reduces the large increase to some extent in the Delay circuit compared to the Basic circuit.

To address the role of plasmid size/copy number variation and translational efficiency between the Basic and Delay circuits, we have constructed a Control Delay circuit (TC2G, see Material & Methods) and checked our induction kinetics (see Supporting Information S1 section B, Fig. S3). The kinetics of induction of the Control Delay circuit is similar to the Basic circuit at different inducer concentrations. Hence, it is clear that both plasmid size difference and translational efficiency of later cistrons are not the major contributing factors to the increased gene expression (Overshoot) in the Delay circuit. The experimental results (Fig. 4) follows the theoretical curves (Fig. 2) to show that both the Basic, as well as, the Delay circuit shows overshoot, which is larger in the case of the Delay circuit. This feature of “overshoot” in temporal gene expression between the Delay and the Basic circuit is, therefore, primarily due to the delay in repression. Theoretically the steady state values of both are predicted to be the same, but the experimental Delay circuit (C2TG) did not reach the same steady state level as that of TG for reasons mentioned earlier.

### 2. Delay-induced overshoot and intra-population heterogeneity in gene expression

It is now well established that individual cells in a population can differ significantly in their response to environmental stimuli, which is advantageous in fluctuating environments [5], [35], [45], [56], [57]. Here we show, both theoretically and experimentally that, compared to the Basic negatively auto-regulated circuit, the Delay circuit has a large heterogeneity in gene expression among the individual cells within a population.

#### (a) Theoretical studies with model circuits

**Figure 5:**
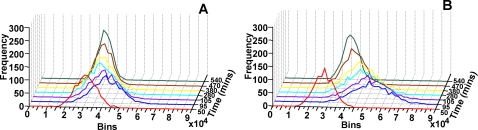
Theoretical frequency distributions of GFP in cell populations: (A) Basic, and (B) Delay circuits at different time intervals.

Using our model cell populations (of 1000 cells) with the Basic and Delay circuits having plasmid variation (copy number: 50±10, normally distributed), we determined the distribution of the GFP expression levels in the cells at different time points. The frequency distributions of the GFP fluorescence in the model Basic and Delay circuits are shown in Fig. 5(A) and (B), respectively, where the X-axis shows GFP distribution (bin values), Y-axis the time points, and Z-axis the frequency (number of cells). The large overshoot in GFP expression in Delay circuit is clear from the larger distance the distributions traverse in X-axis (Fig. 5(B)), which eventually returns to the same steady state as in the Basic circuit (Fig. 5(A)). The distributions of the Delay circuit populations also show a broader spread at the initial time points as compared to the Basic circuit. However, this increased heterogeneity is a transient phenomenon, and eventually both the Basic and the Delay circuits reach similar steady state distributions. Thus, the model Delay circuit is predicted to show transient overshoot and increased heterogeneity in gene expressions within the population, when compared to the cell population with the Basic circuit.

#### (b) Experimental studies

**Figure 6:**
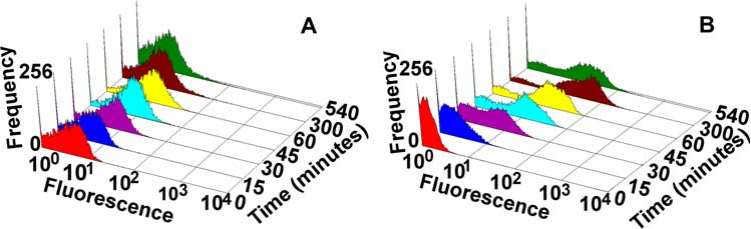
Frequency distribution of GFP fluorescence in experimental cell populations: (A) Basic (TG) and (B) Delay (C2TG) circuits. X- axis: Fluorescence in arbitrary units; Y-axis Time; Z-axis: Frequency.

The GFP fluorescence in 10,000 cells in populations of Basic (TG) and Delay (C2TG) circuits were studied at different time points, after induction with 25 ng/ml Doxycycline, using Flow Cytometry (Fig. S6 in Supporting Information S1 section C). Keeping in view of the transient dynamics of the circuits, Fluorescence Activated Cell Sorter (FACS) measurements were taken at closer time points initially, and then at longer intervals. Figure 6(A) and (B) show the GFP fluorescence distributions in the Basic (TG) and Delay (C2TG) circuits, respectively. There are three features to be noted–(i) presence of bimodality in distribution in the Delay circuit (Fig. 6(B)); (ii) In comparison to the Basic circuit in Fig. 6(A), the population of cells with Delay circuit in Fig. 6(B) show a significantly higher fluorescence in time, which later return to lower levels; and, (iii) A broader distribution of fluorescence in the cell population with Delay circuit.

#### (i) Bimodality in Delay circuit

**Figure 7:**
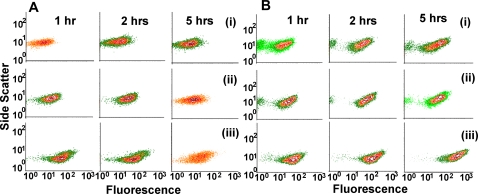
Contour plots of GFP fluorescence distribution: At 1, 2 and 5 hrs after induction with different inducer concentrations in cell populations of (A) TG, and (B) C2TG. Inducer concentrations-(i) 25 ng/ml, (ii) 50 ng/ml, and (iii) 75 ng/ml, of Doxycyline.

For studying the bimodal distribution of fluorescence in the cell populations in Fig. 6(B), a careful and direct comparative analysis with the Basic and Delay circuits at different induction levels–(i) 25 ng/ml, (ii) 50 ng/ml, and (iii) 75 ng/ml of Doxycycline– were done, which is shown in the FACS contour plots in Fig. 7(A) and (B). In general, the cells with the Basic circuit (Fig. 7(A)) shows lower but increasing fluorescence and more homogeneous behaviour at all induction levels, when compared to the Delay circuit in Fig. 7(B). Figure 7(B) clearly shows that the subpopulation of cells with low fluorescence at lower induction level (in row (i)) actually reduces (to the extent of almost vanishing) at increasingly higher induction levels, thereby indicating that the subpopulation of cells with low fluorescence observed at lower induction level (25 ng/ml) is the fraction of un-induced cells. This is seen clearly separated from the induced cells as they show considerably higher GFP expression (“overshoot”) compared to the Basic (TG) circuit (Fig. 7(A)). Thus, the presence of bimodality in Delay circuit cell populations (Fig. 7(B)), which is induced at 25 ng/ml, is a consequence of, but not an inherent property of, the delay element in the circuit. Removal of this low-expressing fraction of cells by gating (Fig. S8 in Supporting Information S1 section C) shows that C2TG continues to have a greater spread than TG.

It may be noted that there is no role of inducer concentration in the models, and hence all cells in both model circuits are induced equally, and their GFP increase with time in Fig. 5(A) and (B).

#### (ii) Delay-induced overshoot of gene expression

**Figure 8:**
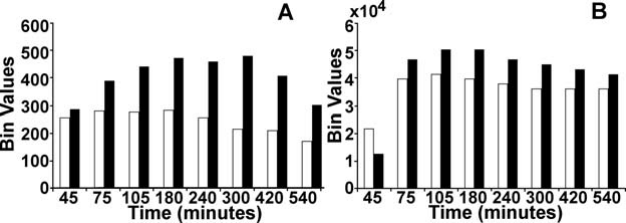
Modal bin values of GFP distribution with time: A) Experimental, and B) Theoretical distributions for the Basic (TG–*white bar*) and Delay (C2TG–*Black bar*) circuits.

Figure 8(A) shows the time course of the modal fluorescence bin values of the gated cells from Fig. 6(A) FACS data, where the white and black bars represent the cell populations with the Basic and Delay circuits, respectively. The model cell distribution for the corresponding circuits (Fig. 5) are shown in Fig. 8(B). Clearly, both the model as well as experimental cell populations with the Delay circuit shows a higher expression level than the Basic circuit. Thus, the overshoot that we observed in the population average measurements (Fig. 4) in the Delay circuit, is also observed at the single cell level. This indicates that delay in repression is responsible for the induction of transiently higher gene expression.

#### (iii) Heterogeneity of gene expression in cells of a population

**Figure 9:**
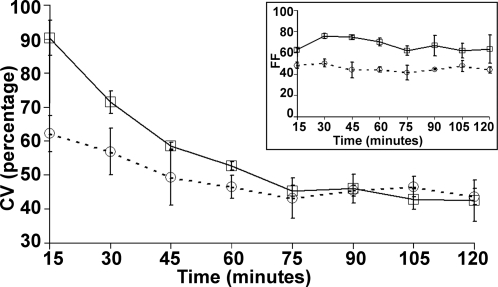
Comparison of Coefficient of Variation (CV) of experimental GFP distributions: Basic (TG-*dashed line with circle*) and Delay (C2TG-*solid line with square*) circuits. Inset: Changes in the Fano Factor (FF) for both the circuits. Error bars are of one standard deviation from three experiments.

A broader distribution of GFP fluorescence is observed in the Delay circuit cell population compared to the Basic circuit. To make a comparative quantification of the intra-population cellular heterogeneity in gene expression in the Basic and Delay circuit at different time points, we plot in Figure 9 the Coefficient of Variation (CV) of their GFP distributions from Fig. 6 (FACS data). The inset shows the corresponding changes in the Fano Factor (FF) for the two circuits. Both these parameters are common measures of assessing variability (noise) in the system (5).

Considering the fact that the mean protein levels are higher in the Delay circuit populations (Fig. 8(A)), its higher CV in Figure 9 clearly shows that variability in the Delay circuit population is considerably higher compared to the Basic circuit during the time of the build-up of the overshoot (till 90 minutes). Additionally, the initial decreasing trend in CV in both the circuits (till about 90 minutes) indicates that, as time increases the intrinsic noise levels reduce in both circuits, (considerably more in the Delay circuit), due to the establishment of the repression in gene expression. The Fano Factor (Figure 9 Inset), on the other hand, continues to show a difference between the two circuits and remains almost constant with time. These two noise strength measures provide evidence that the Delay circuit certainly exhibits greater variability compared to the Basic circuit, which is due to the effect of delayed repression.

**Figure 10:**
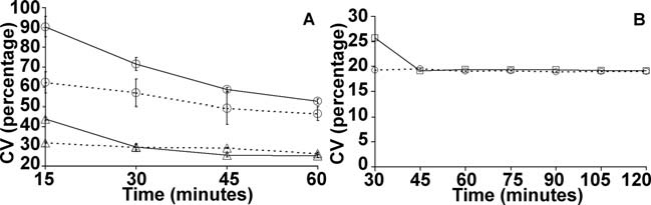
Coefficient of Variation for both Basic (dashed lines) and Delay (solid lines) circuits: A) Experimental populations for different inducer concentrations (25 ng/ml*-circles,* and 75 ng/ml*-triangles*) till 60 min from three experiments, and B) Theoretical simulation (Basic: *dashed line* and Delay: *solid line).*

The model Delay and Basic circuits do not predict any significant difference in their CV over time except at an early time point (see Figure 10(B)). We show from Figure 10(A) that this prediction is consistent with our experimental results. It may be useful to remember that all cells in the theoretical circuit are induced equally and fully. Figure 10(A) shows CV for both Basic (dashed lines) and Delay (solid lines) circuits obtained from the FACS data for increasing inducer concentrations (25 and 75 ng/ml of Doxycycline) for the initial 60 minutes. It may be noted that the Delay circuit at 75 ng/ml induction shows a small difference only at the initial time point and is quite close to what one observes in the model circuits (Fig. 10(B)). Thus the absence of variability in the minimal model of the circuits can be explained. This further gives a proof of concept of our hypothesis that delay in repression is the primary factor for inducing increased inter-cellular heterogeneity in gene expression in a population.

## Discussion

The complex regulation of metabolic and transcriptional networks in the cell is brought about by the interaction of simpler regulatory structures. Study of these simpler structures using theoretical modelling and experimental gene circuit construction provide us with important clues regarding the function of larger, more complex networks. The advantage of this approach is the greater freedom available to dissect out and study specific properties of the regulatory structure of interest in isolation. It allows experimental testing of theoretical predictions, made using mathematical models of the regulation, more easily. The negative auto-regulatory motif has been well studied using this approach, since it is one of the simplest and ubiquitous control structure found in nature [11], [27], [29], [34], [58].

The design adopted by us in the Basic and Delay circuits is a common motif in negative regulation, predominantly observed in biosynthetic pathways [59], where the regulator gene (repressor/co-repressor) in multi-gene, negatively auto-regulated operons, is either the first of many genes, or, one of the last genes separated by other genes from the promoter/operator complex. Depending on where the repressor is situated with respect to the promoter/operator complex, this may lead to differential time delays in repressor production. We classified few naturally occurring negatively auto-regulated operons in *E. coli*, obtained from public databases (Regulondb: http://regulondb.ccg.unam.mx/ and Biocyc: http://biocyc.org/) into “*Delay”* and “*Basic*” type, depending on the position of the repressor gene with respect to its promoter, as shown in Table 1. A *Delay* or *Basic* type is termed if the repressor is the first gene, or, is not the first gene in the operon. In Table 1, when compared with respect to few characteristics, such as, function, length, and number of regulators, both types of operons showed some systematic differences. The Delay-type operons are commonly involved in carbon metabolism and are regulated by multiple regulators and are regulated via multiple promoters, whereas, the Basic-type operons are more diverse, both in the functions they perform, as well as, in the number of regulators that control them. This indicates that there might be some functional relevance to these different commonly observed arrangements of negative auto-regulation. It is also remarkable that in the case of the Basic-type operons, where immediate repression should occur, there are several of them, which regulate the functions of other operons. This could be to ensure that the quantity of these regulators is more tightly controlled, and does not vary much from the steady state. This also suggests the possibility that such control may be employed to ensure that the overshoot occurs in a controlled fashion. In comparison to the naturally occurring lengths of delay, which can range up to nearly 6 KB, our experimental circuit (C2TG) has a delay length of 1.5 KB, which is a conservative one.

**Table 1:** A survey of negatively auto-regulated operons in *E coli*.

Sl. No.	Property	Delay-type	Basic-type
1	Total number of operons	10	10
2	Length of operons (range)	1265 to 5868 bp	1038 to 6747 bp
3	Length of delay (range)	819 to 4921 bp	Not Available
4	Number of operons with multiple promoters	6	3
5	Number of operons with multiple regulators	10	4
6	Number of operons with more levels of regulation (e.g. attenuation)	5	2
7	Number of operons which regulate other operons	4	7
8	Functions of the operons	carbon metabolism------6	1
		global regulation---------1	1
		stress response-----------0	2
		toxin removal------------0	3
		others----------------------3	3

The numerous sequences of biochemical reactions that underlie the complexities of expressing a single gene points towards the fact that the characteristics of such delay may be essential in understanding whole-genome regulation. Time delays involved in operons, such as, the *lac*, *trp* or the *gal* operons have been extensively studied to estimate the time involved in basic biological processes such as transcription and translation [60]–[62]. Recently, it was also shown elegantly that promoters of genes involved in unbranched, multi-step pathways were arranged such that promoters controlling earlier genes in the pathway are turned on faster and produce greater amount of product than later genes [63]. This helps to optimise the energy resources of the cell such that, a protein is not produced much before the actual requirement for it arises. In this paper, we have addressed the issue of time delay in transcriptional feedback directly and simply, and compared it with the case of immediate transcriptional repression.

Delayed feedback strongly affects the dynamics of genetic networks-it can show transient behaviour, or transition to a new fixed point accompanied by oscillations. Theoretical analysis of three eukaryotic genetic regulatory networks showing oscillations have been attributed to a common design of a negative feedback loop with time delay underlying them [32]. Pulse generation has been shown in other gene circuits constructed with different aims, and involving different mechanisms, e.g., by a quorum sensing mechanism, [53], and in a positive transactivation system with delay [33]. We have shown that delay in the establishment of negative feedback is the cause of transient instability, which can be a simple mechanism for generating a pulse of protein through the overshoot mechanism that is observed even in the absence of forced population synchrony [as in 53]. This “Overshoot”, therefore, is a robust phenomenon that is observed at the population level in spite of the noisy nature of the intracellular environment. This simple design principle, in addition to being available “just in time” for metabolic pathways to complete its course [63], possibly uses the Overshoot feature to have enough enzymes left even after loss due to dilution by cell growth and degradation. Thus, such regulator organisation seems to have an advantage by allowing a transient uninhibited gene expression initially for multi-step processes whose end product is required by the cellular processes. Overshoots could be produced by other mechanisms as well. It has been shown that when an externally repressed circuit is induced, an overshoot of transcriptional response is observed in response to steady levels of inducer, which then falls back to its pre-induction level (due to pumping out of the inducer by multi-drug efflux pumps), rendering the cell resistant to further induction [64]. This has been observed at the single cell level only, and disappears at the population average level. Whereas, the phenomenon we observe in the Delay circuit persists both at single cell and population levels.

The interesting observation in our work is the presence of larger heterogeneity in gene expression in the cells with the Delay circuit compared to the Basic negative auto-regulatory case. In the Basic circuit, we observe a more homogeneous distribution of GFP in the cell populations that gradually moves from a low fluorescence value to higher ones with increasing inducer concentration. In the Delay circuit, on the other hand, at intermediate inducer values, fluorescence distribution is spread wider, and some cells seem not to express GFP at all. A single population is seen only at high inducer concentration representing all induced cells. Similar bimodality has also been observed for gene expression without negative feedback at medium inducer concentration and intermediate repression values [11]. Recent studies [11], [65] have suggested that the noise in negative auto-regulatory circuits, such as the one analysed here, should mainly come from fluctuations in plasmid numbers. We have kept the extent of plasmid variation similar in the circuits (Basic, Control Delay, and Delay), and hence the role of external noise due to this feature should be similar. Yet the increased variability in the Delay circuit clearly shows that this heterogeneity in the gene expression is due to the delayed repression. One possible reason could be that both the extrinsic and intrinsic noise are observed in an enhanced manner in the Delay circuit population during the period of unregulated gene expression (after induction and before repression sets in). The reduction in the coefficient of variation with time in both circuits indicates that this noise is suppressed due to establishment of repression.

Interestingly, our theoretical model circuits are a minimal representation of the real cells, and they have no consideration of the realistic details such as, cell size or, variation of cell size during growth. It also considers full induction of all cells from the beginning. Our simulation results clearly predict what the experimental circuits would do at high induction levels, viz, the variability in both circuits would reduce, indicating that the heterogeneity in gene expression in the cell population occurs primarily during the unregulated state at lower induction level, which finally returns to the same level as the basic circuit, albeit delayed, due to establishment of repression. This also points to the fact that all heterogeneity, that is attributed to noise alone, need not be so, as delayed negative auto-regulation can also contribute to population variability in gene expression. Our results add to the growing body of evidence regarding the importance of transient dynamics in bringing about variability that in some cases may aid in the choice of alternate cell fates [66], [67].

The generic origin of delay in biochemical pathways (transcription regulatory pathways or metabolic pathways) implies that there is a high likelihood that the two properties shown in our study-transient overshoot and generation of heterogeneity in gene expression in cell population-play an important role in gene regulation. This motif of regulation can do two things successfully even before the stabilising effect of repression sets in. The overshoot allows for gene products being available in large amount for multi-step pathways to function, and it can also act as a dominant source of large deterministic variability paving way to increase the phenotypic diversity of a population of cells before the negative regulation sets in. It has been documented that phenotypic diversity can be crucial for many processes [5], [35], [57], [68]. Our theoretical and experimental results, thus, provide important clues and give possible rationale for delayed feedbacks to be such a generic feature in gene organisations in cells.

## Materials and Methods

### Construction of the Gene Circuits

The different circuits (transcriptional units) were constructed and cloned in *E.coli* DH5α as described in Supporting Information S1 section A, with the unstable green fluorescence protein (GFP) as the reporter. All components (promoter, repressor, and spacer DNA as the delay element) used for construction were external to the genomic DNA to avoid direct genomic interference.

### Design of the circuits

The design of our negative auto-regulatory transcriptional modules with and without delay is given in Fig. 1. The Basic circuit, TG, consists of the tetR gene and the reporter gene (gfp) after the promoter (pLtet-01). In the Delay circuit, C2TG, two copies of cI gene from λ phage is inserted before the repressor gene so that the production of the repressor is delayed, thus leading to a delay in the establishment of repression. All the proteins-TetR, GFP and CI are tagged with degradation tags (‘deg’ in Fig. 1) to decrease their half-lives in the cell [69]. In both the circuits, the arrangement of the genes is in the form of an “Artificial Operon”, such that independent proteins are produced from a common transcript (transcription stop shown as ‘term’ in Fig. 1). An additional Control Delay circuit (TC2G) is constructed in which the position of the repressor is the same as in TG, but the position of the reporter is identical to that in C2TG, i.e., GFP is the fourth cistron in this circuit with two copies of cI gene as the Delay element. This circuit, then, is identical to the Delay circuit (C2TG) in length, number of cistrons, and position of the Reporter gene, except for the position of the repressor, TetR.

On induction by Doxycycline (inducer), gene expression ensues, and fluorescence increases. Depending on the promoter strength, the number of plasmid copies, the half-life of the repressor, and the concentration of inducer, a dynamic balance is achieved between the intracellular inducer molecules and the repressor, and repression is re-established. The detailed study of growth of the circuits TG and C2TG show similar behaviour (Fig. S1 and Fig. S2 in Supporting Information S1 section B). The induction kinetics of TG (Basic), C2TG (Delay), and TC2G (Control Delay) circuits (Fig. S3 in Supporting Information S1 section B) shows similar trends of expression of the Basic and Control Delay circuits, and large overshoot with the Delay circuit at all inducer concentrations. Also, the expression kinetics of TetR (determined using SDS-PAGE, Fig. S4 and Fig. S5 in Supporting Information S1 section B) shows that TetR expression does follow that of GFP in these circuits. We fixed on an inducer concentration of 25 ng/ml for our studies since the growth of the circuits was minimally affected at this concentration, though the Delay circuit did start showing retarded growth from 7 hr after induction.

### Population average measurements

The fluorescence of a representative population (200 µl) was measured in live cells using a Fluorescence ELISA reader (Spectramax, Molecular Devices). The settings used were 491 nm absorbance, 535 nm emission, with 530 nm auto cut off to reduce background fluorescence. The instrument sensitivity was kept at maximum and an average of 6 measurements was taken. As controls for auto fluorescence, uninduced samples of each induced circuit were also measured at each time point. The auto fluorescence was subtracted from the induced fluorescence and normalised by the growth (measured as OD at 600 nm) of the circuit to give the final fluorescence value per unit time.

### GFP measurement in individual cells in the population

Flow cytometry was carried out using a FACS Calibur or a FACS Vantage (Becton-Dickenson) with a 488 nm laser using fixed cells as described in Supporting Information S1 section A. Fluorescence data was collected using logarithmic amplification in the FL1 channel. The cells were threshold on Forward Scatter (log amplified) to exclude dust particles. A total of 10,000 events were collected per sample. For comparison of heterogeneity, triplicate samples of all the circuits were analysed on the same day to avoid problems due to day-to-day variations while using logarithmic amplification. Uninduced controls for each circuit and a positive control with constitutive GFP expression were also run and were used to set the gain such that both highly expressing and non-expressing cells could be detected and represented on the same scale. The cells were then gated according to size to obtain a more homogenous population as discussed in Supporting Information S1 section A, and shown in Fig. S6 (Supporting Information S1 section C). This procedure did not affect the fluorescence distribution of the population as can be seen in Fig. S7 (Supporting Information S1 section C and also from Fig. S8).

### Modelling Methods

Deterministic and Stochastic models of the gene circuits corresponding to the negatively auto-regulated pathways with and without delay in repression were constructed using coupled differential equations and the modified Gillespie Algorithm [44], [70]. The delay is modelled by assuming a delay in the production of the repressor protein (due to the increased length of the transcript preceding the repressor). A basal delay has been introduced in the Basic circuit to mimic the multi-step process of gene expression. The detail description of the models and the parameter values used are given in Supporting Information S2 and Table S2. The deterministic four-variable model includes GFP as a separate variable, since the experimental measurements have been done of the reporter (GFP). To study the transient dynamics in this model with delayed repression, stability analysis of the steady state using the Nyquist criteria were performed [71].

It is known that variation in plasmid copy number in populations of cells is an important source of variability [11], [65], hence, for modelling individual cell behaviour in a population, we used it as a source of population heterogeneity, and constructed the stochastic model for studying the temporal expression of the gene circuits. In this approach, the plasmid copy number was varied in 1000 individual ‘cells’ (or runs) based on a value derived from the normal distribution with mean 50 and standard deviation 10. The distribution of GFP levels at different time points for 1000 cells were determined The results thus obtained are comparable to the results of FACS experiments to determine the population heterogeneity. We compared the variability in the populations of two circuits by two common measures of variability a) Coefficient of Variation, CV = (standard deviation/mean)*100, and b) Fano Factor, FF = variance/mean of the GFP distribution for both theoretical and experimental data.

## Supporting Information

Supporting Information S1 Construction of circuits; Cell Culture; Fixing protocol; Growth and Induction kinetics of different circuits; Gating of the FACS data.
(0.04 MB DOC)

Supporting Information S2 Models, Methods and Parameters
(0.05 MB DOC)

Figure S1 Growth of the circuits with and without induction. Legend: Uninduced-TG: triangles with solid lines, C2TG: Black circles with dotted lines. Induced-TG: squares with solid lines, C2TG: diamonds with dotted lines.
(0.04 MB TIF)

Figure S2 Growth of the circuits at different inducer concentrations (a) 25 ng/ml, (b) 50 ng/ml and (c) 75 ng/ml of Doxycycline. (Squares, TG and Circles, C2TG).
(0.05 MB TIF)

Figure S3 Kinetics of GFP fluorescence of the Basic (TG, triangles), Control Delay (TC2G, circles), and Delay (C2TG, squares) circuits upon induction in four independent experiments at different inducer concentrations-(a) 25, (b) 50, and (c) 75 ng/ml of Doxycycline, at 1 hr interval.
(0.35 MB TIF)

Figure S4 SDS-PAGE results showing TetR kinetics at different time points after induction-(A) Basic (TG) and (B) Delay (C2TG) circuits. The arrow indicates the position of the TetR band. The lower panel in each figure indicates the control band.
(0.10 MB TIF)

Figure S5 Quantification of intensity of the bands (Triangles, TG and circles, C2TG).
(0.03 MB TIF)

Figure S6 Gating of cell populations according to size, showing the greater uniformity of cell size in the gated population as compared to ungated population. (A) and (C) for TG and C2TG populations at 0 hr before gating, and, (B) and (D) for TG and C2TG populations after gating.
(0.14 MB TIF)

Figure S7 The effect of gating on the fluorescence distribution of the cells. TG (A) ungated and (B) gated; C2TG (C) ungated and (D) gated. X- axis = different time points represented in serial numbers, Y-axis = percentage of cells (white bars = R1; horizontal bars = R2 ; black bars = R3).
(0.70 MB TIF)

Figure S8 Frequency distribution of the gated population at various time points after induction of the circuits (A) TG and (B) C2TG. The X- axis: fluorescence in arbitrary units; Y-axis Time in min; Z-axis: Frequency.
(0.53 MB TIF)

Figure S9 Kinetics of TetR in the Basic (solid circles with dashed lines) and the Delay (squares with solid lines) circuits for (A) deterministic model and (B) stochastic model (average of 100 simulations).
(0.13 MB TIF)

Table S1 Comparison of kc value with k values from Nyquist loci for increasing delay.
(0.02 MB DOC)

Table S2 Parameter values used for deterministic and stochastic simulations
(0.02 MB DOC)

## References

[1] Thieffry D, Huerta AM, Perez-Rueda E, Collado-Vides J (1998). From specific gene regulation to genomic networks: a global analysis of transcriptional regulation in *Escherichia coli*.. Bioessays.

[2] Shen-Orr S, Milo R, Mangan S, Alon U (2002). Network motifs in the transcriptional regulation network of *Escherichia coli* .. Nat Genet.

[3] Milo R, Itzkovitz S, Kashtan N, Levitt R, Shen-Orr S (2004). Superfamilies of evolved and designed networks.. Science.

[4] Alon U (2007). Network motifs: theory and experimental approaches.. Nat Rev Genet.

[5] Kaern M, Elston TC, Blake WJ, Collins JJ (2005). Stochasticity in gene expression: from theories to phenotypes.. Nat Rev Genet.

[6] Smolen P, Baxter DA, Byrne JH (2000). Mathematical modeling of gene networks.. Neuron.

[7] Kepler TB, Elston TC (2001). Stochasticity in transcriptional regulation: origins, consequences and mathematical representations.. Biophys J.

[8] Becskei A, Kaufmann BB, van Oudenaarden A (2005). Contributions of low molecule number and chromosomal positioning to stochastic gene expression.. Nat Genet.

[9] Colman-Lerner A, Gordon A, Serra E, Chin T, Resnekov O (2005). Regulated cell-to-cell variation in a cell-fate decision system.. Nature.

[10] Hooshangi S, Weiss R (2006). The effect of negative feedback on noise propagation in transcriptional gene networks.. Chaos.

[11] Dublanche Y, Michalodimitrakis K, Kümmerer N, Foglierini M, Serrano L (2006). Noise in transcription negative feedback loops: simulation and experimental analysis.. Mol Syst Biol.

[12] Maithreye R, Sinha S (2007). Propagation of extrinsic perturbation in a negatively auto-regulated pathway.. Phys Biol.

[13] Levine E, Hwa T (2007). Stochastic fluctuations in metabolic pathways.. Proc Natl Acad Sci USA.

[14] Ptashne M (2004). A Genetic Switch: Phage Lambda Revisited. 3rd Ed..

[15] Gardner TS, Cantor CR, Collins JJ (2000). Construction of a genetic toggle switch in *Escherichia coli*.. Nature.

[16] Tyson JJ (1991). Modeling cell division cycle: cdc2 and cyclin interactions.. Proc Natl Acad Sci USA.

[17] Bar-Or RL, Maya R, Segel LA, Alon U, Levine AJ (2000). Generation of oscillations by the p53-Mdm2 feedback loop: a theoretical and experimental study.. Proc Natl Acad Sci USA.

[18] Roenneberg T, Merrow M (2003). The network of time: understanding the molecular circadian system.. Curr Biol.

[19] Becskei A, Seraphin B, Serrano L (2001). Positive feedback in eukaryotic gene networks: cell differentiation by graded to binary response conversion.. EMBO J.

[20] Ozbudak EM, Thattai M, Lim HN, Shraiman BI, van Oudenaarden A (2004). Multistability in the lactose utilization network of *Escherichia coli*.. Nature.

[21] Maeda YT, Sano M (2006). Regulatory dynamics of synthetic gene networks with positive feedback.. J Mol Biol.

[22] Van Hoek MJA, Hogeweg P (2006). *In silico* evolved *lac* operons exhibit bistability for artificial inducers but not for lactose.. Biophys J.

[23] Maamar H, Raj A, Dubnau D (2007). Noise in gene expression determines cell fate in *Bacillus subtilis*.. Science.

[24] Sureka K, Ghosh B, Dasgupta A, Basu J, Kundu M (2008). Positive feedback and noise activate the stringent response regulator Rel in Mycobacteria.. PLoS ONE.

[25] Goodwin BC (1965). Oscillatory behavior of enzymatic control processes.. Adv Enzyme Regul.

[26] Sinha S, Ramaswamy R (1988). Complex behaviour of the repressible operon.. J Theor Biol.

[27] Becskei A, Serrano L (2000). Engineering stability in gene networks by auto-regulation Nature.

[28] Elowitz MB, Leibler S (2000). A synthetic oscillatory network of transcriptional regulators.. Nature.

[29] Rosenfeld N, Elowitz MB, Alon U (2002). Negative autoregulation speeds the response times of transcription networks.. J Mol Biol.

[30] Mangan S, Zaslaver A, Alon U (2003). The coherent feedforward loop serves as a sign-sensitive delay element in transcription networks.. J Mol Biol.

[31] Monk N (2003). Oscillatory expression of Hes1, p53, and NF-κB driven by transcriptional time delays.. Curr Biol.

[32] Tiana G, Krishna S, Pigolotti S, Jensen MH, Sneppen K (2007). Oscillations and temporal signalling in cells.. Phys Biol.

[33] Weinberger LS, Shenk T (2007). An HIV feedback resistor: Auto-regulatory circuit deactivator and noise buffer.. PLoS Biol.

[34] Savageau MA (1974). Comparison of classical and autogenous systems of regulation in inducible operons.. Nature.

[35] Rao CV, Wolf DM, Arkin AP (2002). Control, exploitation and tolerance of intracellular noise.. Nat Genet.

[36] McAdams HH, Arkin A (1997). Stochastic mechanisms in gene expression.. Proc Natl Acad Sci USA.

[37] Austin DW, Allen MS, McCollum JM, Dar RD, Wilgus JR (2006). Gene network shaping of inherent noise spectra.. Nature.

[38] Simpson ML, Cox CD, Sayler GS (2003). Frequency domain analysis of noise in autoregulated gene circuits.. Proc Natl Acad Sci USA.

[39] Struhl K (1999). Fundamentally different logic of gene regulation in eukaryotes and prokaryotes. Cell.

[40] Rosenfeld N, Alon U (2003). Response delays and the structure of transcription networks.. J Mol Biol.

[41] Rapp PE (1975). A theoretical investigation of a large class of biochemical oscillators.. Math Biosc.

[42] Maithreye R, Sinha S, Deutsch A, Falcke M, Howard J, Zimmermann W (2003). Modelling of simple biochemical pathways.. Function and regulation of cellular systems: Experiments and Models. Birkhauser.

[43] Gopalsamy K (1992). Stability and oscillations in delay differential equations of population dynamics..

[44] Bratsun D, Volfson D, Tsimring LS, Hasty J (2005). Delay-induced stochastic oscillations in gene regulation.. Proc Natl Acad Sci USA.

[45] Ozbudak EM, Thattai M, Kurtser I, Grossman AD, van Oudenaarden A (2002). Regulation of noise in the expression of a single gene.. Nat Genet.

[46] Swain PS, Elowitz MB, Siggia ED (2002). Intrinsic and extrinsic contributions to stochasticity in gene expression.. Proc Natl Acad Sci USA.

[47] Blake WJ, Kaern M, Cantor CR, Collins JJ (2003). Noise in eukaryotic gene expression.. Nature.

[48] Raser JM, O'Shea EK (2004). Control of stochasticity in eukaryotic gene expression.. Science.

[49] Chen L, Wang R, Zhou T, Aihara K (2005). Noise-induced cooperative behavior in a multicell system.. Bioinformatics.

[50] Hooshangi S, Thiberge S, Weiss R (2005). Ultrasensitivity and noise propagation in a synthetic transcriptional cascade.. Proc Natl Acad Sci U S A.

[51] Pedraza JM, van Oudenaarden A (2005). Noise propagation in gene networks.. Science.

[52] Banerjee B, Balasubramanian S, Ananthakrishna G, Ramakrishnan TV, Shivashankar GV (2004). Tracking operator state fluctuations in gene expression in single cells.. Biophys J.

[53] Basu S, Mehreja R, Thiberge S, Chen MT, Weiss R (2004). Spatiotemporal control of gene expression with pulse-generating networks.. Proc Natl Acad Sci U S A.

[54] Freedman HI, Gopalsamy K (1986). Global stability in time-delayed single species dynamics.. Bull Math Biol.

[55] Niculescu S (1998). Stability and hyperbolicity of linear systems with delayed state: a matrix-pencil approach.. IMA J of Math Control & Information..

[56] McAdams HH, Arkin A (1999). It's a noisy business! Genetic regulation at the nanomolar scale.. Trends Genet.

[57] Losick R, Desplan C (2008). Stochasticity and cell fate.. Science.

[58] Thieffry D, Thomas R (1995). Dynamical behaviour of biological regulatory networks- II. Immunity control in bacteriophage lambda.. Bull Math Biol.

[59] Nelson DL, Cox MM (2007). Lehninger Principles of Biochemistry..

[60] Alpers D, Tomkins G (1966). Sequential transcription of the genes of the lactose operon and its regulation by protein synthesis.. J Biol Chem.

[61] Goldberger R, Berberich M (1965). Sequential repression and derepression of enzymes for his biosynthesis in *Salmonella typhimurium*.. Proc Nat Acad Sci USA.

[62] Starlinger P (1967). Sequential appearance of galactose enzymes in *Escherichia coli*.. Mol Gen Genet.

[63] Zaslaver A, Mayo AE, Rosenberg R, Bashkin P, Sberro H (2004). Just-in-time transcription program in metabolic pathways.. Nat Genet.

[64] Le TT, Emonet T, Harlepp S, Guet CC, Cluzel P (2006). Dynamical determinants of inducible gene expression in a single bacterium.. Biophys J.

[65] Paulsson J (2004). Summing up the noise in gene networks.. Nature.

[66] Shin D, Lee EJ, Huang H, Groisman EA (2006). A positive feedback loop promotes transcription surge that jump-starts Salmonella virulence circuit.. Science.

[67] Suel GM, Garcia-Ojalvo J, Liberman LM, Elowitz MB (2006). An excitable gene regulatory circuit induces transient cellular differentiation.. Nature.

[68] Raser JM, O'Shea EK (2005). Noise in gene expression: origins, consequences, and control.. Science.

[69] Andersen JB, Sternberg C, Poulsen LK, Bjørn SP, Givskov M (1998). New unstable variants of green fluorescent protein for studies of transient gene expression in bacteria.. Appl Environ Microbiol.

[70] Gillespie DT (1977). Exact stochastic simulation of coupled chemical reactions.. J Phys Chem.

[71] Segel L (1980). Mathematical models in molecular and cellular biology..

