# Correction: Delay-Induced Transient Increase and Heterogeneity in Gene Expression in Negatively Auto-Regulated Gene Circuits

**DOI:** 10.1371/annotation/5d7be1dd-cef8-499b-bcf4-a79d69245fa7

**Published:** 2008-10-08

**Authors:** R. Maithreye, Ram Rup Sarkar, Veena K. Parnaik, Somdatta Sinha

There was an error in the data of Table 1. Row 3 of the last column should read "Not Applicable."

