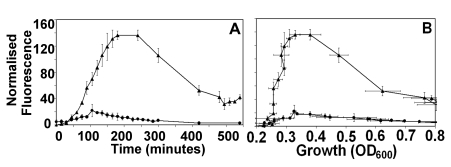# Correction: Delay-Induced Transient Increase and Heterogeneity in Gene Expression in Negatively Auto-Regulated Gene Circuits

**DOI:** 10.1371/annotation/8243fdb8-7945-4edc-b7bd-18a56445ed57

**Published:** 2008-10-08

**Authors:** R. Maithreye, Ram Rup Sarkar, Veena K. Parnaik, Somdatta Sinha

The x-axis labels of Figure 4B are incorrect. Please view the corrected figure here: 

**Figure:**