# Supplementary material for: Delay-Induced Transient Increase and Heterogeneity in Gene Expression in Negatively Auto-Regulated Gene Circuits
Source: PLoS One. 2008 Aug 13;3(8):e2972. doi: 10.1371/journal.pone.0002972 (PMC2494610; doi:10.1371/journal.pone.0002972)
Supplement: Supporting Information S1 — Construction of circuits; Cell Culture; Fixing protocol; Growth and Induction kinetics of different circuits; Gating of the FACS data. (0.04 MB DOC) [file pone.0002972.s001.doc]

# Supporting Information S1

**Section A**

**Construction of circuits**: The basic negative autoregulatory module (without delay) is similar to the one studied by other workers (Becksei and Serrano, 2000; Rosenfeld et al, 2002) with a few modifications. The genes were obtained from the following sources: the ssrA degradation tagged genes cI_de_ and tetR_de_ were from the repressilator (Elowitz and Leibler, 2000) and gfp+ was obtained from Dr. Niederweiss (Scholz et al, 2000) to which an ssrA degradation tag was added (Keiler et al, 1996; Andersen et al, 1998). The circuits were constructed by sequential cloning and confirmed by DNA sequencing. The genes were first sub cloned into high-copy pBSSK vectors (Stratagene) and finally assembled into the lower copy pZE vector (Lutz and Bujard, 1997) for expression studies. The pZE vector possesses a pLtet-01 promoter and a transcription terminator in between which all the constructs were cloned so that they express as a single transcript but produce separate proteins. All the constructs had the same RBS and translational start and stop sequences to minimise variation in the efficiency of protein synthesis.

**Cell Culture:** *E. coli* strain DH5α (λ-, recA1-) was used for cloning as well as expression analyses. The cells were transformed with the different plasmids and were grown in Minimal A medium supplemented with 0.1% Casamino acids and 0.1% glycerol and containing 25 μg/ml of Kanamycin at 37°C and induced with 25 ng/ml of Doxycyline (Sigma Aldrich). In all the experiments, the cells were inoculated into liquid media overnight and diluted to a final OD_600_ of 0.1 for the experiments. The cells were induced in mid-log phase at 0.2 OD_600_ and measurements were continued for 9 hours, with measurements at 15-minute intervals for the first 3 hours after induction and measurements at 1-hour intervals subsequently.

**Fixing protocol:** A modified version of the protocol given in Chen et al (1999) for fixing of GFP in *Escherichia coli* was followed. Cells to be fixed were spun down at 3000 rpm for 2 min, resuspended in 200 to 400 μl of 1X PBS and mixed with 2X fixative (2X fixative 4.8% paraformaldehyde, 0.03% gluteraldehyde in 1X PBS). The cells were allowed to stand at RT for 15 minutes followed by a 30 minute incubation on ice after which 3 washes in 1X PBS were given and cells were stored in 1X PBS at 4°C. Cells can be stored for up to 2 months without significant loss of signal. For all flow cytometry studies, the cells were analysed within two weeks of fixing.

**Section B**

## Growth and Induction kinetics of different circuits

**Growth kinetics of induced and uninduced circuits:** Growth of the basic (TG) and the delay (C2TG) circuits with and without induction at 25ng/ml of Doxycycline are shown in Fig. S1.

**Growth and kinetics of circuits at different inducer concentrations:**

**Growth** of the Basic and the Delay circuits at different inducer concentrations are given in Fig. S2(a-c). It can be seen that the growth of the circuit C2TG is slower than that of TG at all inducer concentrations. The Control Delay circuit (TC2G) had similar growth kinetics as Delay circuit.

**Induction kinetics** of the Basic, Control Delay, and Delay circuits at different inducer concentrations are given in Fig. S3(a-c). The circuit C2TG shows higher levels of fluorescence at all inducer concentrations than the basic circuit TG, and the control delay circuit (TC2G).

**Determination of TetR kinetics on induction:**

Here we show that GFP faithfully represents the kinetics of the repressor, TetR.

**Protocol for SDS-PAGE measurements:** For analysing the expression of TetR over time, the proteins expressed from the circuits were subjected to SDS-PAGE analysis. Protein was extracted from equal number of cells (equivalent to an OD of 0.3 in 1.5 ml) both before induction and at various time points after induction with 75 ng/ml Doxycycline and was run on a 15% SDS- PAGE gel to ensure maximum separation of the bands. After the run, the gel was stained using Coomassie Brilliant Blue (Sigma Aldrich), destained and photographed. The photograph was scanned at a resolution of 300 dpi and bands were quantified using the Gene Tools software (Syngene) as follows:

The band corresponding to the TetR protein was quantified at all time points after induction as well as in the uninduced sample. As a control, a band whose intensity did not change with induction was quantified (Fig. S4(A-B)). The uninduced TetR protein value was subtracted from the induced values; normalised by the control, and plotted versus time (Fig. S5).

**Section C**

**Gating of the FACS data**: FACS data were gated according to size, as shown in Fig. S6, to obtain a more homogeneous population in terms of cell size for fluorescence analysis. The parameters for gating were set such that maximum number of data points in all the samples is covered. The following protocol was used: a) The Forward scatter (FSC) versus Side scatter (SSC) dot plots of the FACS samples were plotted; b) A rectangular region from ~ 3 to 60 units on the SSC scale and a similar range on the FSC scale was chosen; c) The cells inside this region were gated out and used in the analysis of their fluorescence distribution.

To determine whether this procedure alters the fluorescence distribution, the cells in the ungated as well as the gated samples were divided into three groups depending on their levels of expression - R1: from 1-10 units of fluorescence; R2: from 10 to 100 units of fluorescence; and R3: from 100 to 1000 units of fluorescence. The fractions of the cell population were quantified in these three groups and shown in Fig. S7, which shows that the trend of the data was not altered by the gating procedure; the ungated and the gated populations showed similar behaviour in both the circuits.

**Frequency distribution after removal of low-expressing cells**: A threshold of ~3 units of fluorescence was set such that the low-expressing subpopulation is removed from the C2TG population at all time points. The cells expressing above this threshold were then considered to get a new frequency distribution for both the TG and the C2TG populations. This new distribution was normalised to a frequency of 256 for both the populations and replotted as shown in Fig. S8.

**References:**

1. Andersen JB, Sternberg C, Poulsen LK, Bjørn SP, Givskov M, et al. (1998) New unstable variants of green fluorescent protein for studies of transient gene expression in bacteria. Appl Environ Microbiol 64: 2240-2246.

2. Becskei A, Serrano L (2000) Engineering stability in gene networks by auto-regulation. Nature 405: 590–593.

3. Chen JC, Weiss DS, Ghigo JM, Beckwith J (1999) Septal localization of FtsQ, an essential cell division protein in Escherichia coli. J Bacteriol 181: 521-530.

4. Elowitz MB, Leibler S (2000) A synthetic oscillatory network of transcriptional regulators. Nature 403: 335–338.

5. Keiler K, Waller P, Sauer R (1996) Role of a peptide tagging system in degradation of proteins synthesized from damaged messenger RNA. Science 271: 990-993.

6. Lutz R, Bujard H (1997) Independent and tight regulation of transcriptional units in Escherichia coli via the LacR/O, the TetR/O and AraC/I1-I2 regulatory elements. Nucleic Acids Res 25: 1203-1210.

7. Rosenfeld N, Elowitz MB, Alon U (2002) Negative autoregulation speeds the response times of transcription networks. J Mol Biol 323: 785–793.

8. Scholz O, Thiel A, Hillen W, Niederweis M (2000) Quantitative analysis of gene expression with an improved green fluorescent protein. Eur J Biochem 267: 1565-1570.
