# Supplementary material for: Delay-Induced Transient Increase and Heterogeneity in Gene Expression in Negatively Auto-Regulated Gene Circuits
Source: PLoS One. 2008 Aug 13;3(8):e2972. doi: 10.1371/journal.pone.0002972 (PMC2494610; doi:10.1371/journal.pone.0002972)
Supplement: Supporting Information S2 — Models, Methods and Parameters (0.05 MB DOC) [file pone.0002972.s002.doc]

# Supporting Information S2

***Models, Methods and Parameters:***

**Deterministic model**: The four variables considered to model the two circuits are concentrations of the mRNA or the transcript (*m*), the repressor, TetR (*p*), the reporter, GFP (*f*), and the available free promoters (*g*). For simplicity, the degradation rates of *m*, *p* and *f* are assumed to follow first order kinetics. Considering these factors, the model is:

$$\begin{matrix} \frac{dm}{dt}=\beta_{1}g-\alpha_{1}m \\ \frac{dp}{dt}=\beta_{2}m_{(t-\tau)}-\alpha_{2}p \\ \frac{df}{dt}=\beta_{2}m_{(t-\tau)}-\alpha_{3}f \\ \frac{dg}{dt}=k_{2}(g_{t}-g)-k_{1}gp \end{matrix}$$

(1)

where, τ is the time delay on the production of the repressor and reporter from the common mRNA; α_1_, α_2_ and α_3_ are degradation rates of the mRNA, TetR and GFP; β_1_ and β_2_ are the rates of transcription and translation; *k_1_* and *k_2_* are the association and dissociation rates for the promoter-repressor complex reaction; and *g_t_* is the total number of promoters. For our theoretical studies, we have used a basal delay of 3 minutes (Rosenfeld et al, 2002) in Basic circuit and an additional delay of 1 minute for the Delay circuit.

**Stability analysis using Nyquist Criteria:** From the Routh-Hurwitz criterion of the no-delay system (τ = 0), we found that there exists a critical value for the gain constant k, say k_c_, such that the system is stable if k < k_c_ and unstable if k > k_c_, where,

$$k_{c}=\frac{(\alpha_{1}+\alpha_{2})(k_{2}+k_{1}p^{*})(\alpha_{1}+\alpha_{2}+k_{2}+k_{1}p^{*})+\alpha_{1}\alpha_{2}(\alpha_{1}+\alpha_{2})}{k_{1}\alpha_{1}\alpha_{2}p^{*}}$$

$p*=\frac{-B+\sqrt{B^{2}-4AC}}{2A}$ $f*=\frac{\alpha_{2}}{\alpha_{3}}p*$ $m*=\frac{\alpha_{2}}{\beta_{2}}p*$ $g*=\frac{\alpha_{1}\alpha_{2}}{\beta_{1}\beta_{2}}p*$and, , , , and , are the steady

state solutions of the system with $A=k_{1}\alpha_{1}\alpha_{2}$, $B=k_{2}\alpha_{1}\alpha_{2}$ and $C=-k_{2}\beta_{1}\beta_{2}g_{t}$.

At k = k_c_ the roots are purely imaginary and oscillations result. For (k + ε) < k_c_ (ε being an arbitrarily small positive number), the oscillations damp out. Table S1 shows that the feedback gain (k), for all delays (0-500 sec) considered, are less than the critical value k_c_. The Nyquist loci, G(iw), for the transfer function (Segel, 1980) of the model is shown in Fig. 3 (in Main Text) for a range of delay time.

**Stochastic model**: The following molecular reactions are considered in the stochastic model to describe the negative auto-regulatory system.

β_1_

1. g mRNA Transcription

β_2_

2. mRNA TetR Translation

β_2_

3. mRNA GFP Translation

k_1_

4. g + TetR [gTetR] Repressor binding

k_2_

5. [gTetR] g + TetR Repressor dissociation

α_1_

6. mRNA ϕ Degradation

α_2_

7. TetR ϕ Degradation

α_3_

8. GFP ϕ Degradation

Here, φ represents the degradation products of mRNA, TetR and GFP, and the other parameters are as described in the deterministic model.

To represent the time delay in the stochastic case, a modified Gillespie’s Algorithm (Gillespie, 1977; Bratsun et al, 2005) was used, where the protein products (TetR and GFP) are incremented only after the delay. The kinetics of GFP, for both the deterministic and the stochastic models of Basic and Delay circuits (as shown in Fig. 2 in the main text), follow the TetR kinetics as shown in Fig. S9.

**Parameter Set:** To choose the parameters of the model to reflect the experimental circuits, we used a strong promoter, a multi-copy plasmid in our constructs, and increased the turn-over of the repressor and the reporter by addition of degradation tags to the proteins.. The parameters are obtained from the literature (Bundschuh et al, 2002; Elowitz and Leibler, 2000; Andersen et al, 1998; Lutz and Bujard, 1997) and are given in Table S2.

**References:**

1. Andersen JB, Sternberg C, Poulsen LK, Bjørn SP, Givskov M, et al. (1998) New unstable variants of green fluorescent protein for studies of transient gene expression in bacteria. Appl Environ Microbiol 64: 2240-2246.

2. Bratsun D, Volfson D, Tsimring LS, Hasty J (2005) Delay-induced stochastic oscillations in gene regulation. Proc Natl Acad Sci (USA) 102: 14593-14598.

3. Bundschuh R, Hayot F, Jayaprakash C (2003) The role of dimerization in noise reduction of simple genetic networks. J Theor Biol 220: 261-269.

4. Elowitz MB, Leibler S (2000) A synthetic oscillatory network of transcriptional regulators. Nature 403: 335–338.

5. Gillespie DT (1977) Exact stochastic simulation of coupled chemical reactions. J Phys Chem 81: 2340-2361.

6. Lutz R, Bujard H (1997) Independent and tight regulation of transcriptional units in Escherichia coli via the LacR/O, the TetR/O and AraC/I1-I2 regulatory elements. Nucleic Acids Res 25: 1203-1210.

7. Rosenfeld N, Elowitz MB, Alon U (2002) Negative autoregulation speeds the response times of transcription networks. J Mol Biol 323: 785–793.

8. Segel L (1980) Mathematical models in molecular and cellular biology. Cambridge: Cambridge University Press. 786p.
