# Supplementary figures and images for: Delay-Induced Transient Increase and Heterogeneity in Gene Expression in Negatively Auto-Regulated Gene Circuits
Source: PLoS One. 2008 Aug 13;3(8):e2972. doi: 10.1371/journal.pone.0002972 (PMC2494610; doi:10.1371/journal.pone.0002972)

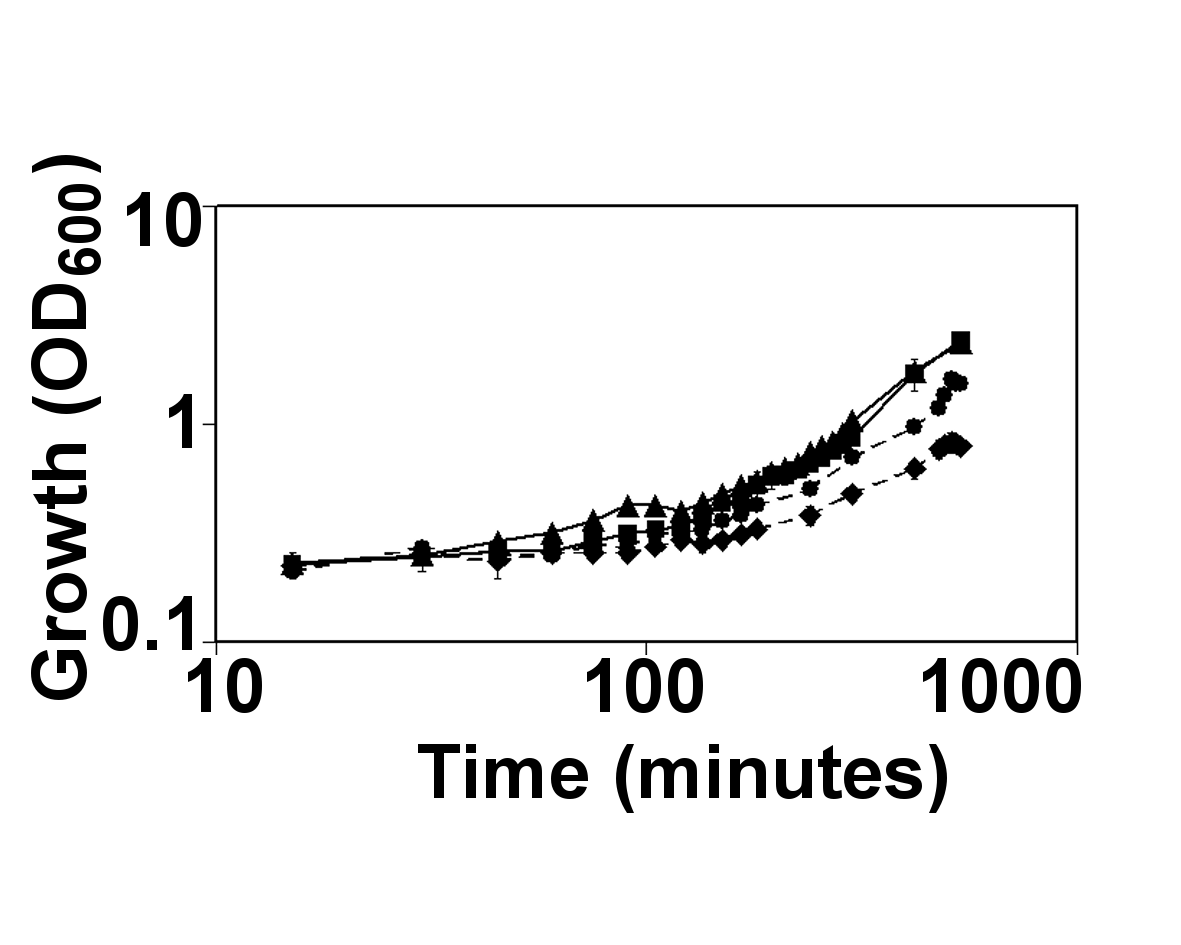

Supplement: Figure S1 — Growth of the circuits with and without induction. Legend: Uninduced-TG: triangles with solid lines, C2TG: Black circles with dotted lines. Induced-TG: squares with solid lines, C2TG: diamonds with dotted lines. (0.04 MB TIF) [file pone.0002972.s003.tif]

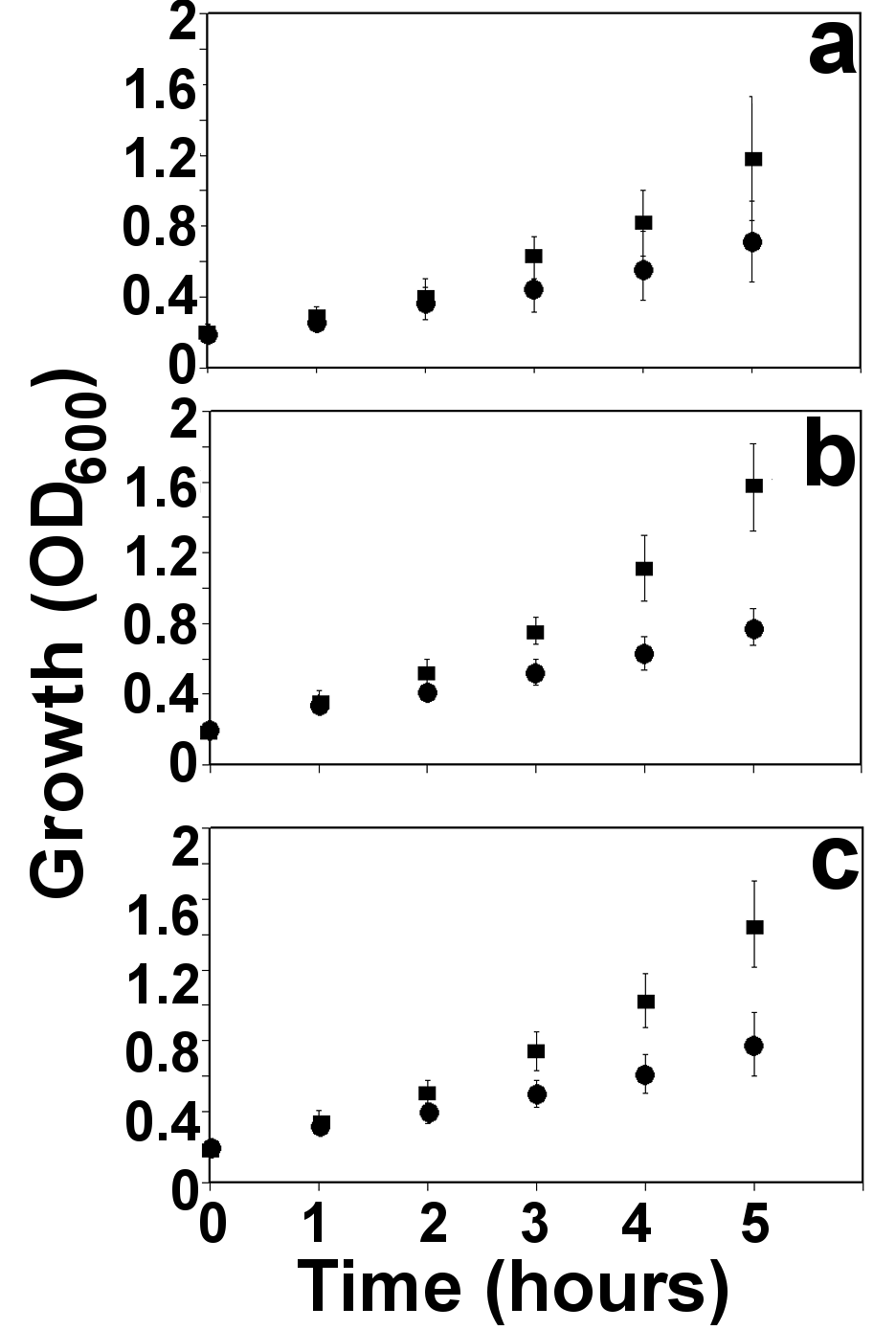

Supplement: Figure S2 — Growth of the circuits at different inducer concentrations (a) 25 ng/ml, (b) 50 ng/ml and (c) 75 ng/ml of Doxycycline. (Squares, TG and Circles, C2TG). (0.05 MB TIF) [file pone.0002972.s004.tif]

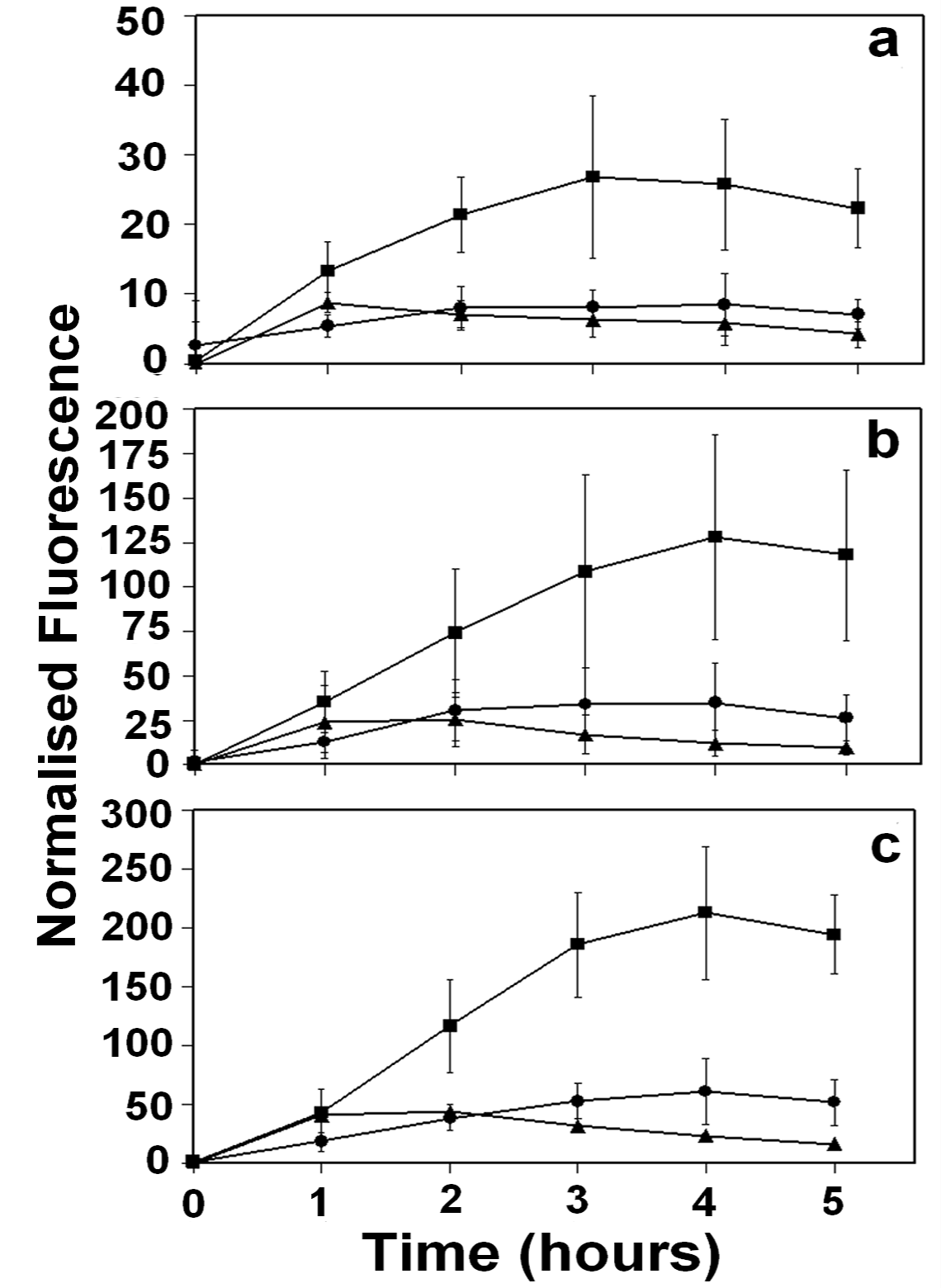

Supplement: Figure S3 — Kinetics of GFP fluorescence of the Basic (TG, triangles), Control Delay (TC2G, circles), and Delay (C2TG, squares) circuits upon induction in four independent experiments at different inducer concentrations-(a) 25, (b) 50, and (c) 75 ng/ml of Doxycycline, at 1 hr interval. (0.35 MB TIF) [file pone.0002972.s005.tif]

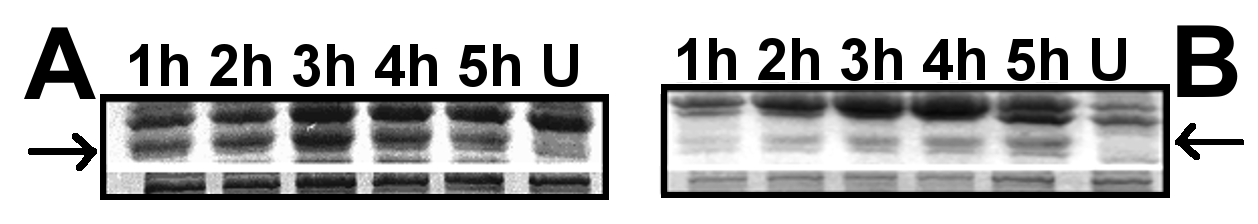

Supplement: Figure S4 — SDS-PAGE results showing TetR kinetics at different time points after induction-(A) Basic (TG) and (B) Delay (C2TG) circuits. The arrow indicates the position of the TetR band. The lower panel in each figure indicates the control band. (0.10 MB TIF) [file pone.0002972.s006.tif]

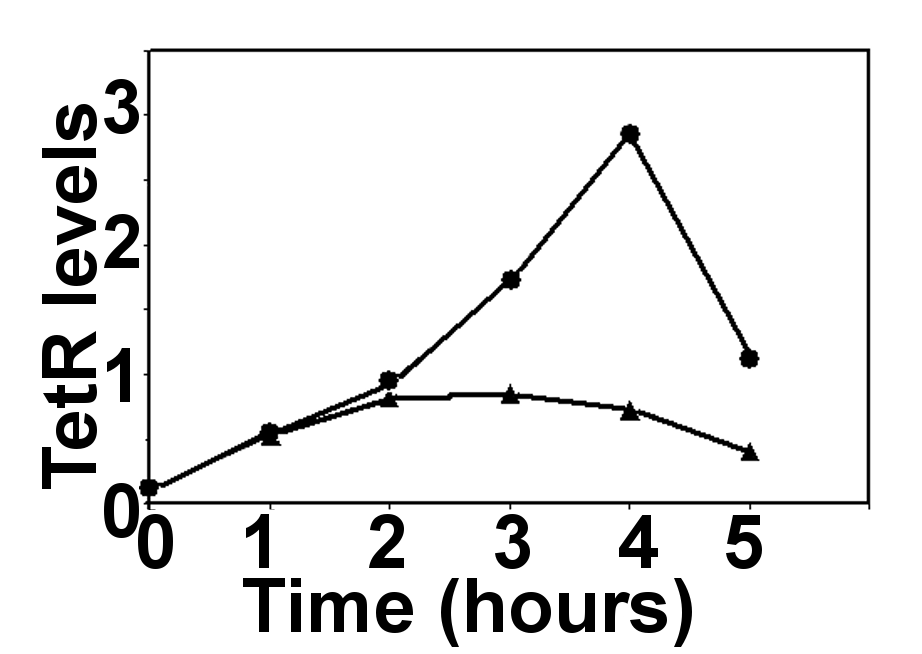

Supplement: Figure S5 — Quantification of intensity of the bands (Triangles, TG and circles, C2TG). (0.03 MB TIF) [file pone.0002972.s007.tif]

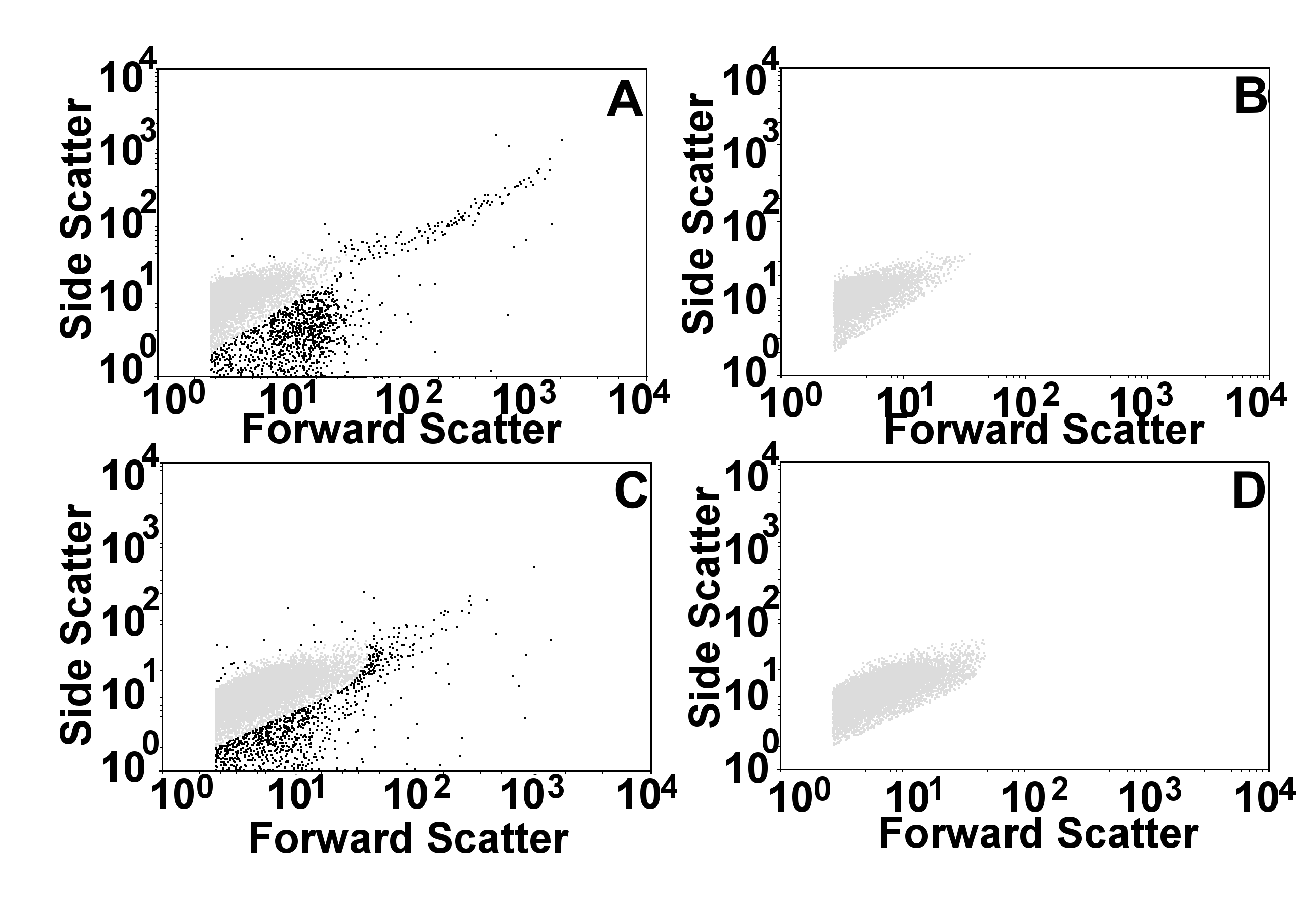

Supplement: Figure S6 — Gating of cell populations according to size, showing the greater uniformity of cell size in the gated population as compared to ungated population. (A) and (C) for TG and C2TG populations at 0 hr before gating, and, (B) and (D) for TG and C2TG populations after gating. (0.14 MB TIF) [file pone.0002972.s008.tif]

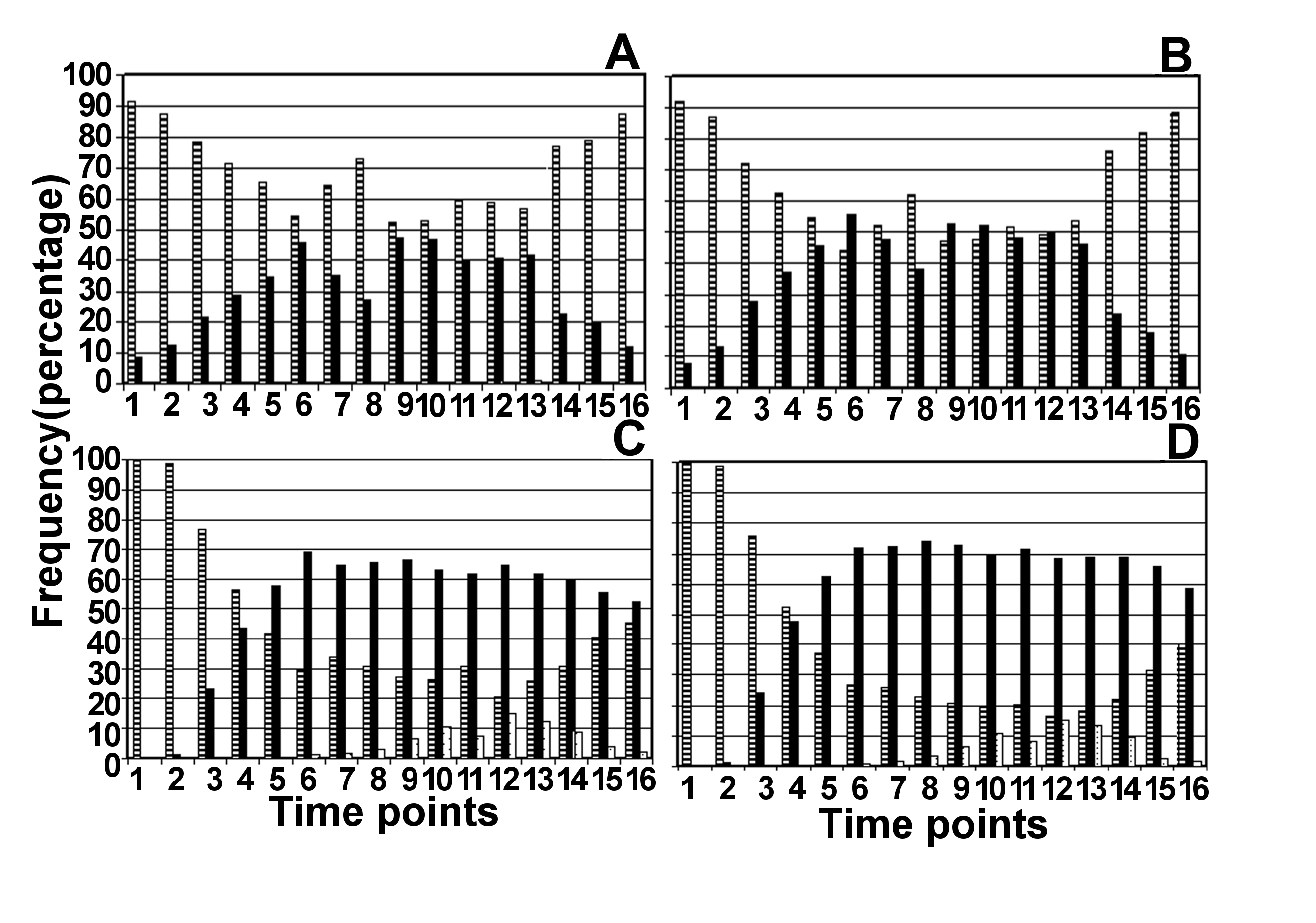

Supplement: Figure S7 — The effect of gating on the fluorescence distribution of the cells. TG (A) ungated and (B) gated; C2TG (C) ungated and (D) gated. X- axis = different time points represented in serial numbers, Y-axis = percentage of cells (white bars = R1; horizontal bars = R2 ; black bars = R3). (0.70 MB TIF) [file pone.0002972.s009.tif]

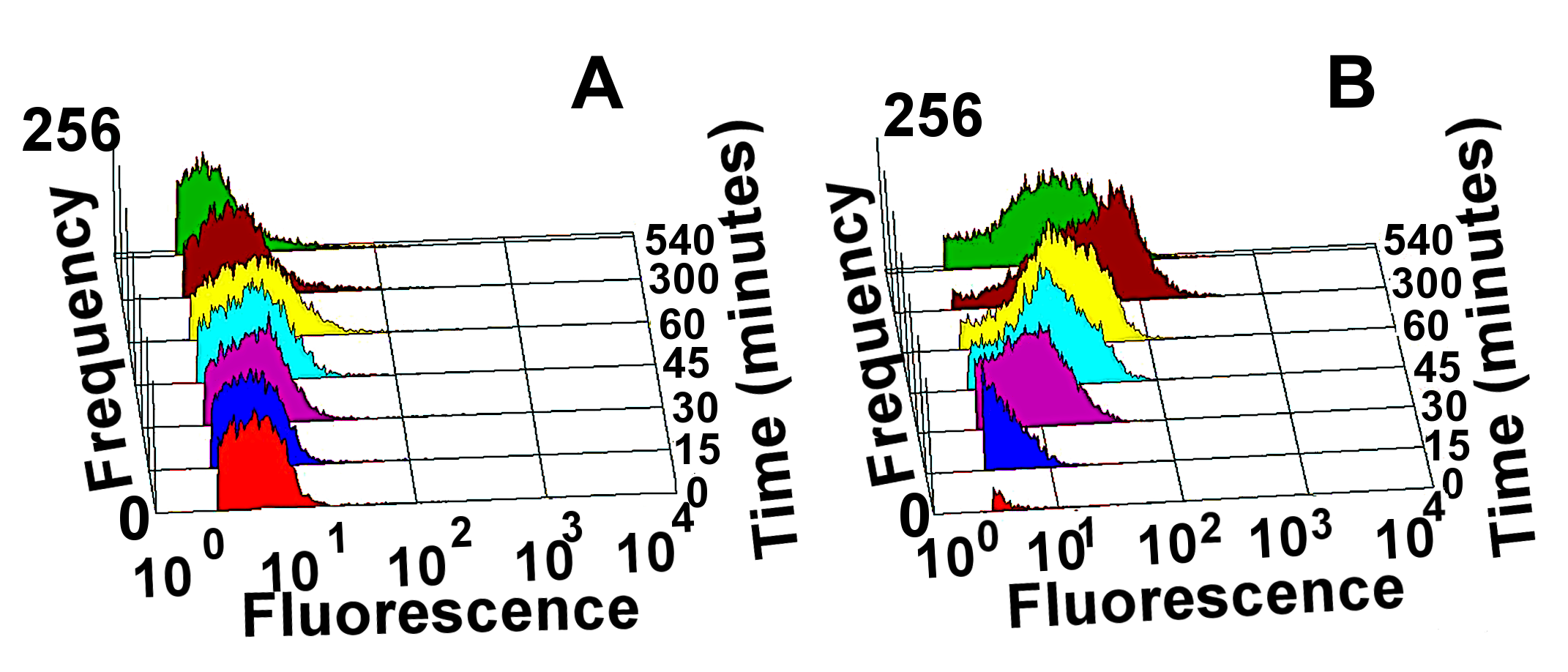

Supplement: Figure S8 — Frequency distribution of the gated population at various time points after induction of the circuits (A) TG and (B) C2TG. The X- axis: fluorescence in arbitrary units; Y-axis Time in min; Z-axis: Frequency. (0.53 MB TIF) [file pone.0002972.s010.tif]

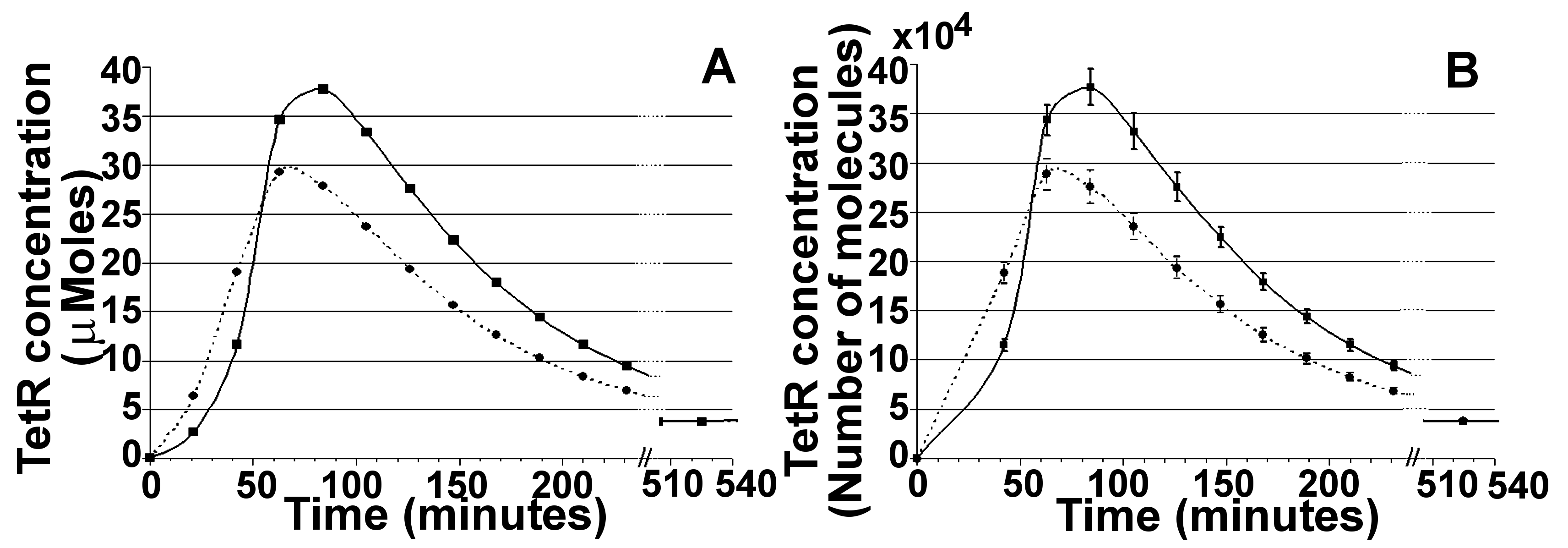

Supplement: Figure S9 — Kinetics of TetR in the Basic (solid circles with dashed lines) and the Delay (squares with solid lines) circuits for (A) deterministic model and (B) stochastic model (average of 100 simulations). (0.13 MB TIF) [file pone.0002972.s011.tif]
