# Supplementary material for: Delay-Induced Transient Increase and Heterogeneity in Gene Expression in Negatively Auto-Regulated Gene Circuits
Source: PLoS One. 2008 Aug 13;3(8):e2972. doi: 10.1371/journal.pone.0002972 (PMC2494610; doi:10.1371/journal.pone.0002972)
Supplement: Table S1 — Comparison of kc value with k values from Nyquist loci for increasing delay. (0.02 MB DOC) [file pone.0002972.s012.doc]

***Table S1:*** *Comparison of k_c_ value with k values from Nyquist loci for increasing delay.*

| **Critical Value** | **Time delays (τ)** | **k values calculated from Fig. 3**  **(k = -1/value of x-intersection)** |
| --- | --- | --- |
| k_c_ = 49615 | 20 | 55.86 |
|  | 40 | 28.82 |
|  | 60 | 19.65 |
|  | 500 | 3.22 |
