# Supplementary material for: Delay-Induced Transient Increase and Heterogeneity in Gene Expression in Negatively Auto-Regulated Gene Circuits
Source: PLoS One. 2008 Aug 13;3(8):e2972. doi: 10.1371/journal.pone.0002972 (PMC2494610; doi:10.1371/journal.pone.0002972)
Supplement: Table S2 — Parameter values used for deterministic and stochastic simulations (0.02 MB DOC) [file pone.0002972.s013.doc]

***Table S2:*** *Parameter values used for deterministic and stochastic simulations*

| **Parameter** | **Deterministic Value** | **Stochastic Value** |
| --- | --- | --- |
| β_2_ | 4.3 x10^-2^ sec^-1^ | 4.3x10^-2^ sec^-1^ |
| β_1_ | 0.6 x10^-9^M sec^-1^ | 0.6 molecules sec^-1^ |
| α_1_ | 5.7 x10^-3^sec^-1^ | 5.7x10^-3^ sec^-1^ |
| α_2_ | 1.15 x10^-3^ sec^-1^ | 1.15 x10^-3^ sec^-1^ |
| α_3_ | 1.28 x10^-4^sec^-1^ | 1.28 x10^-4^ sec^-1^ |
| k_1_ | 1.2 x 10^+7^M ^-1^sec^-1^ | 0.012 molecules ^-1^sec^-1^ |
| k_2_ | 0.9 sec^-1^ | 0.9 sec^-1^ |
| g_t_ | 50 | 50 |
